# **Synopsis of the species of *Ortholinea*** **Shulman, 1962 (Cnidaria: Myxosporea: Ortholineidae)**

**DOI:** 10.1007/s11230-024-10155-2

**Published:** 2024-05-03

**Authors:** Luis F. Rangel, Sónia Rocha, Maria J. Santos

**Affiliations:** 1 Interdisciplinary Centre of Marine and Environmental Research (CIIMAR/CIMAR), Laboratory of Animal Parasitology and Pathology, University of Porto, Matosinhos, Portugal; 2 Laboratory of Animal Parasitology and Pathology, Biology Department, Faculty of Sciences (FCUP), University of Porto, Porto, Portugal; 3 Instituto de Investigação e Inovação em Saúde (i3S), University of Porto, Porto, Portugal; 4 Institute of Biomedical Sciences Abel Salazar (ICBAS), University of Porto, Porto, Portugal

## Abstract

A synopsis of *Ortholinea* Shulman, 1962 (Cnidaria: Myxosporea: Ortholineidae) is presented and identifies 26 nominal species presently allocated within this genus. Species morphological and morphometric features, tissue tropism, type-host, and type-locality are provided from original descriptions. Data from subsequent redescriptions and reports is also given. Accession numbers to sequences deposited in GenBank are indicated when available, and the myxospores were redrawn based on original descriptions. The information gathered shows that *Ortholinea* infect a wide taxonomic variety of freshwater and marine fish. Nonetheless, the broad host specificity reported for several species is not fully supported by morphological descriptions and requires molecular corroboration. The members of this genus are coelozoic and mainly parasitize the urinary system, with few species occurring in the gallbladder. *Ortholinea visakhapatnamensis* is the only exception, being histozoic in the visceral peritoneum. Molecular data of the small subunit ribosomal RNA gene (SSU rDNA) is available for about one third of *Ortholinea* species, with genetic interspecific variation ranging between 1.65% and 29.1%. Phylogenetic analyses reveal *Ortholinea* to be polyphyletic, with available SSU rDNA sequences clustering within the subclades of the highly heterogenous freshwater urinary clade of the oligochaete-infecting lineage. The life cycles of two *Ortholinea* species have been clarified based on molecular inferences and identify triactinomyxon actinospores as counterparts, and marine oligochaetes of the family Naididae as permissive hosts to this genus.

## Introduction

The class Myxozoa Grassé, 1970 comprises microscopic obligate cnidarian parasites. There are more than 2,200 known myxozoan species, presently distributed among 66 genera and 20 families (Fiala et al., 2015; Freeman & Kristmundsson, 2015; Freeman et al., 2020). The subclass Myxosporea Bütschli, 1881 is the most diversified, encompassing species characterized by a complex life cycle that require annelids (oligochaetes, polychaetes, and sipunculids) as definitive hosts, and vertebrates (usually fish, but also birds, reptiles, and mammals) as intermediate hosts (Lom & Dyková, 2006). The family Ortholineidae Lom & Noble, 1984 is particularly heterogenous, comprising three coelozoic genera that parasitize marine fish - *Ortholinea* Shulman, 1962, *Neomyxobolus* Chen & Hsieh, 1960 and *Kentmoseria* Lom & Dyková, 1995 - but also, two histozoic genera parasitizing freshwater fish - *Cardimyxobolus* Ma, Dong & Wang, 1982, and *Triangula* Chen & Hsieh, 1984.

The oldest species presently included in the genus *Ortholinea* were originally described as belonging to the genus *Sphaerospora* Thélohan, 1892, family Myxidiidae Thélohan, 1892 (Thélohan, 1892). Davis (1917) considered the latter to be extremely heterogenous and, therefore, instituted the family Sphaerosporidae to include the genera *Sphaerospora* and *Myxoproteus* Doflein, 1898. This author also created the genus *Sinuolinea* to better encompass *Sinuolinea dimorpha* (Davis, 1916), originally described as belonging to the genus *Sphaerospora*. Later, Shulman (1959) instituted the family Sinuolineidae to include the genus *Sinuolinea*, comprising species with myxospores having a sinuous suture line, and the genus *Davisia* Laird, 1953, comprising species with myxospores having lateral processes. Shulman (1962) expanded the family Sinuolineidae with the creation of the genus *Ortholinea* for encompassing species with myxospores having a straight suture line, i.e., *O. divergens* Thélohan, 1895, *O. orientalis* Shulman & Shulman-Albova, 1953 and *O. polymorpha* Davis, 1917 (originally included in the genus *Sphaerospora*). *Ortholinea divergens* was established as type species.

Lom and Noble (1984) created the family Ortholineidae within the also newly established suborder Variisporina, which united the members of the former suborders Bipolarina Tripathi, 1948 emend. Shulman, 1959 and Eurysporea Kudo, 1919 emend. Shulman, 1962. The family Ortholineidae was created to accommodate the genera *Ortholinea* and *Neomyxobolus*, which are coelozoic and develop myxospores with polar capsules that are in the same plane as the suture line, unlike the remaining genera of the family Sinuolineidae, in which the polar capsules are located perpendicular to the suture line (Lom & Noble, 1984). The genera *Cardimyxobolus*, *Triangula* and *Kentmoseria* were later added to the family Ortholineidae, despite the first two differing from all other genera included in this family based on their histozoic development (Lom & Dyková, 2006). Currently, the taxonomic classification of the genus *Ortholinea* is:

Phylum Cnidaria Hatschek, 1888

Class Myxozoa Grassé, 1970

Subclass Myxosporea Bütschli, 1881

Order Bivalvulida Shulman, 1959

Suborder Variisporina Lom & Noble, 1984

Family Ortholineidae Lom & Noble, 1984

Genus *Ortholinea* Shulman, 1962

Type species *Ortholinea divergens* (Thélohan, 1895) Shulman, 1962

Following the creation of the family Ortholineidae, other *Sphaerospora* species were ultimately transferred to the genus *Ortholinea*, namely *Sphaerospora undulans* Meglitsch, 1970 and *Sphaerospora sphaerocapsularae* Wierzbicka 1986 (Arthur & Lom, 1985; Sitjà-Bobadilla & Álvarez-Pellitero, 1994). In turn, three species have been transferred from *Ortholinea* to other genera. *Parvicapsula irregularis* (Kabata, 1962) was originally described as *Sphaerospora irregularis*, and later redescribed by Gaevskaya and Kovaleva (1984) as *Myxoproteus irregularis*. Unknowing of this taxonomic alteration, Arthur and Lom (1985) transferred *S. irregularis* to the genus *Ortholinea*. The validity of this species, however, was questioned by Køie et al. (2007), who suggested it to be more related with *Parvicapsula* Shulman, 1953; an assumption that was ultimately confirmed by Kodádková et al. (2014) through molecular analyses. The species *Triangula perccotti* (Dogiel & Akhmerov, 1960 in Akhmerov, 1960) was also originally described as a member of the genus *Sphaerospora* (Akhmerov, 1960), and soon after transferred to the genus *Myxosoma* by Shulman (1962), who renamed it *M. percotti*, thus dropping one ‘c’ from the specific name. Later, Lom and Noble (1984) proposed the demise of the genus *Myxosoma* and synonymy of its species with *Myxobolus* Bütschli, 1882. However, unknowing of the taxonomic alteration proposed by Shulman (1962), Arthur and Lom (1985) transferred *S. perccotti* to the genus *Ortholinea*. Curiously, Landsberg and Lom (1991) later transferred *Myxosoma percotti* to the genus *Triangula*, and Sokolov (2013) redescribed the myxospores and recovered the ‘c’ of the specific name to rename it *Triangula perccotti*. Lastly, the species *Kentmoseria alata* (Kent & Moser, 1990) was originally described as *Ortholinea alata*, despite its myxospores possessing valvular projections atypical for the genus. Lom and Dyková (1995) considered this feature as being sufficient for creating the genus *Kentmoseria*, in which this species is presently allocated.

Currently, there are 26 species of *Ortholinea*, as listed in Table 1. However, this species list may be expected to change based on the increasing molecular data. For instance, the broad host specificity and morphological variations reported by different accounts of the type species, *O. divergens*, suggest it to be a species complex. Thus, the acquisition of molecular data from infections in the type host and other reported hosts will probably identify new species records. *Ortholinea antipae* Moshu & Trombitsky, 2006 and *Ortholinea clupeidae* Aseeva, 2000 were suggested to be synonyms of *Ortholinea orientalis* (Shulman & Shulman-Albova, 1953) Shulman, 1962 based on myxospore morphological similarity (Karlsbakk & Køie, 2011), however, all three species continued to be named in the literature, probably due to the lack of molecular data confirming this synonymy. The histozoic nature of *O. visakhapatnamensis* Padma Dorothy & Kalavati, 1993 is atypical of the genus *Ortholinea*, so it is likely that a taxonomic revision of this species will occur once molecular data becomes available.

**Table 1:** Myxospore morphometry of *Ortholinea* species. Measures are given in micrometres and, when available, include mean, standard deviation, and range in parentheses. Absence of data is indicated by a hyphen. Abbreviations: F, Figure number; PCL, Polar capsule length (or diameter when width is missing); PCW, Polar capsule width; PTC, Polar tubule coils; SBL, Myxospore length; SBT, Myxospore thickness; SBW, Myxospore width; *) Width and thickness values interchanged in the original description. Data obtained from the original descriptions.

Species	F	SBL	SBW	SBT	PCL	PCW	PTC
*O. africanus*	1	7.7 ± 0.2 (6.9–8.5)	7.7 ± 0.2 (6.9–8.5)	4.1 ± 0.4 (3.8–4.6)	2.9 ± 0.2 (2.3–3.8)	–	4 (or 5)
*O. antipae*	2	6.8–7.5	6.2–7.2	5.0–5.4	1.8–2.5	–	3 (or 4)
*O. asymmetrica*	3	8.0–10.0	7.5–9.3	–	3.3–4.0	2.0–3.3	–
*O. auratae*	4	9.0 ± 0.3 (8.2–10.1)	8.3 ± 0.4 (7.5–9.1)	7.2 ± 0.5 (6.3–8.4)	3.2 ± 0.1 (2.9–3.6)	2.7 ± 0.1 (2.4–2.9)	3–4
*O. australis*	5	8.7 (7.8–10.4)	8.0 (7.3–9.5)	6.2–7.3	3.7 (2.8–4.4)	2.9 (2.3–3.2)	3–4
*O. basma*	6	13.5 ± 1.0 (12.0–15.0)	12.3 ± 0.5 (11.8–13.0)	–	4.3 ± 0.3 (4.0–4.8)	3.5 ± 0.5 (3.0–4.3)	4–5
*O. clupeidae*	7	7.4–9.5	5.5–6.3	–	2.5–3.0	–	–
*O. concentrica*	8	8.9 ± 0.6 (8.2–11.0)	8.7 ± 0.6 (7.9–11.0)	8.3 ± 0.4 (7.7–9.0)	3.1 ± 0.3 (2.4–3.8)	2.7 ± 0.2 (2.3–3.6)	3–4
*O. divergens*	9	10.0	12.0	–	–	–	–
*O. fluviatilis*	10	8.3 (7.9–8.4)	7.8 (7.3–8.0)	6.8	3.1 (2.8–3.3)	–	4–6
*O. gadusiae*	11	10.8 (9.0–11.7)	9.2 (9.0–9.9)	8.0 (7.2–9.0)	2.8 (1.8–3.0)	2.0 (1.0–2.5)	4–5
*O. gobiusi*	12	7.7–9.8	7.0–7.2	4.8–5.0	1.8–2.1	1.8–2.1	–
*O. indica*	13	7.4 ± 0.8 (6.5–9.0)	6.2 ± 0.5 (5.5–8.0)	–	1.7 ± 0.2 (1.5–2.0)	–	3–5
*O. labracis*	14	7.6 ± 0.3 (6.8–8.7)	7.2 ± 0.2 (6.7–7.7)	6.5 ± 0.4 (5.8–7.7)	3.0 ± 0.2 (2.6–3.4)	2.4 ± 0.1 (2.0–2.9)	4–5
*O. lauquen*	15	7.3 ± 0.4 (6.5−8.3)	7.6 ± 0.4 (6.6−8.8)	7.5 ± 0.6 (6.3−8.8)	3.3 ± 0.3 (2.2−4.0)	2.4 ± 0.3 (1.8−3.1)	3–4
*O. macrouri*	16	9.3–10.6	8.0–9.0	6.5–7.0	3.3–3.9	2.6	–
*O. mullusi*	17	9.3 ± 0.2 (9.0−9.7)	8.7 ± 0.3 (8.2−9.3)	7.7 ± 0.1 (7.5−7.9)	3.1 ± 0.1 (3.0−3.2)	2.5 ± 0.1 (2.4−2.6)	3–4
*O. nupchi*	18	7.6 ± 0.5 (6.4‒9.0)	7.3 ± 0.5 (6.2‒8.5)	6.7 ± 0.3 (6.3‒7.2)	3.1 ± 0.2 (2.6‒3.4)	2.1 ± 0.2 (1.5‒2.4)	3–4
*O. orientalis*	19	7.5–8.5	7.5–7.6	5.1	2.2–2.9	–	–
*O. polymorpha*	20	8.0 (7.0–10.0)	8.0 (7.0–10.0)	–	4.0–5.0	2.0–2.5	–
*O. saudii*	21	10.0 ± 0.4 (9.0–11.0)	12.0 ± 0.5 (11.0–13.0)	–	4.5 ± 0.3 (4.0−5.0)	4.5 ± 0.3 (4.0−5.0)	3
*O. scatophagi*	22	7.3 ± 0.7 (6.2‒8.7)	6.9 ± 0.7 (5.9‒8.2)	6.5 ± 0.4 (6.1‒6.9)	2.6 ± 0.4 (1.7‒3.2)	2.2 ± 0.3 (1.3‒3.0)	4–5
*O. sphaerocapsularae*	23	11.4 ± 0.6 (10.4–12.8)	10.4 ± 0.5 (9.6–12.0)*	(8.0–9.2)*	4.1 ± 0.2 (3.6–4.4)	3.4 ± 0.3 (2.8–3.8)	6
*O. striateculus*	24	10.1 (9.1–10.5)	10.0 (8.9–10.4)	–	3.5 (3.4–3.6)	2.9 (2.8–3.1)	5–7
*O. undulans*	25	8.3 (7.0–10.0)	7.4 (6.0–9.0)	6.3 (5.0–8.0)	2.9 (2.0–4.0)	2.2 (2.0–3.0)	–
*O. visakhapatnamensis*	26	5.9 (5.2–6.0) or 6.9	5.9 (5.2–6.0) or 5.1	–	3.0 (2.6–3.5)	2.2 (1.7–2.6)	5–6

*Ortholinea* species have been described from both freshwater and marine fish worldwide. Based on original descriptions, *Ortholinea* have been recorded from 31 distinct fish species belonging to 25 families and 17 orders. Eight species have been reported from Europe, another 8 from Asia, 4 from Africa, 3 from Australia, 2 from South America, and a single species has been reported from North America. Reports show that this genus is present in the Atlantic, Indian, and Pacific oceans, as well as in the Mediterranean Sea and Black Sea.

The genus typically groups marine species. The few exceptions are *O. fluviatilis* Lom & Dyková, 1995, *O. africanus* Abdel-Ghaffar et al., 2008, *O. lauquen* Alama-Bermejo et al., 2019, and *Ortholinea sphaerocapsularae* (Wierzbicka, 1986) Sitjà-Bobadilla & Álvarez-Pellitero, 1994. The last was originally described from specimens of the European eel *Anguilla anguilla* (Linnaeus) caught from freshwater in Poland. However, the European eel is a catadromous fish that spawns in the Sargasso Sea, with its larvae migrating to European waters carried by ocean currents of the North Atlantic Drift (Wright et al., 2022). Therefore, it is possible that infection by *O. sphaerocapsularae* is acquired in marine or estuarine environments.

Most *Ortholinea* species have been reported from a single fish host. Exceptions are *O. divergens*, reported from a wide array of taxonomically distinct fish hosts; *O. orientalis*, reported from both Clupeiformes and Gadiformes; *O. clupeidae* and *O. undulans*, reported from two distinct families of Clupeiformes and Pleuronectiformes, respectively; *O. australis* Lom, Rohde & Dyková, 1992, reported from two different sparid genera; and *O. polymorpha*, reported from two different batrachoidid species belonging to the genus *Opsanus* Rafinesque, 1818. The molecular data available for *O. orientalis* confirm that this species is not host specific, given that infections could be verified in two different genera of Clupeidae (Karlsbakk & Køie, 2011). This suggests that *Ortholinea* species might be capable of infecting closely related fish hosts, as in the cases of *O. australis* and *O. polymorpha*. The broader host specificity reported for other species is not fully supported by morphological descriptions and requires molecular corroboration. For instance, Karlsbakk & Køie (2011) noticed differences in the dimensions of *O. orientalis* myxospores between Clupeiformes and Gadiformes that are consistent with the existence of a cryptic species. Reports of *O. divergens* from different fish hosts and geographic localities, sometimes atypically from the gall bladder, are accompanied by significant morphological variations that suggests *O. divergens* constitutes a species complex. Considering that the latter is the type species of the genus *Ortholinea*, it is imperative that future studies seek to resolve this complex, by providing a comprehensive morphological and molecular redescription of *O. divergens* from its type host.

*Ortholinea* are coelozoic, with 23 species (88.5%) reported from the organs of the urinary system. The urinary bladder is the organ most frequently infected (69.2%), followed by the kidney (42.3%), the ureters (19.2%), and the urethra (3.8%); with several species being able to develop in more than one of these organs. Three species of *Ortholinea* parasitize the gallbladder (*O. asymmetrica* Kovaleva, Velev & Vladev, 1993, *O. australis* and *O. orientalis*), with *O. orientalis* also occurring in the urinary bladder. *Ortholinea undulans* parasitizes both the urinary system and the oviducts, and *O. visakhapatnamensis* is the only exception to the coelozoic nature of the genus, forming cysts in the visceral peritoneum. Cysts measure up to 1 mm and form disporic pansporoblasts. All other *Ortholinea* species develop mono-, di- to polysporic plasmodia. Polysporic plasmodia are the most frequently observed, but several species also develop mono- or disporic plasmodia simultaneously. Plasmodia have variable shape and size, and display both ectoplasm (hyalin and transparent) and endoplasm (finely granular). The presence of pseudopods, lobes, peripheral projections, or other villi-like projections is also characteristic of the plasmodial membrane of this genus.

Despite the diversity of terms used to describe *Ortholinea* myxospores (spherical, subspherical, rounded, egg-shaped, triangular, subcircular, etc.), these stages are, in fact, restricted to three general shapes in valvular plane: ellipsoid to spherical, sub-oval to oval, and triangular. In sutural plane, myxospores are ellipsoidal to ovoid, and generally less flattened than in the valvular plane. The two polar capsules are symmetric, either being subspherical to spherical or pyriform, and typically open to opposite sides. The reported number of turns of the polar tubule varies between a minimum of 3 turns and a maximum of 7 turns, with 3–5 turns being the most common. The suture line is generally straight and conspicuous, except for *O. fluviatilis* and *O. undulans*, which have more sinuous or undulating suture lines. The valves usually display surface ridges, except for *O. gadusiae* Sarkar, 1999a, 1999b, *O. indica* Sarkar, 1999a, 1999b and *O. saudii* Abdel-Baki et al., 2015, which have smooth valves.

There is few information regarding the pathogenicity of *Ortholinea* species. These parasites appear to be harmless to their vertebrate hosts, given that macroscopic alterations to infected organs, and external signs of disease and/or mortality have never been reported. Thus far, only microscopic damages have been described in reports of *O. australis*, *O. lauquen* and *O. saudii*. *Ortholinea australis* was reported to cause enlargement of the gallbladder and thickening of its wall, leading to the stagnation of bile outflow, and hepatic disorder (Lom et al., 1992). *Ortholinea lauquen* was associated with both physical and pathological changes to the host kidneys, causing cellular necrosis, disintegration of the tubular epithelium, and occlusion of the tubule lumina due to plasmodia adhesion (Alama-Bermejo et al., 2019). The developmental stages of *O. saudii* were also reported to cause obstruction of the kidney tubules (Abdel-Baki et al., 2015).

Values of prevalence of infection by *Ortholinea* vary considerably, with about one third of the known species displaying values that range between 50 and 100%, and the remaining species displaying values lower than 50%. Nonetheless, molecular-based studies suggest that prevalence of infection may be highly underestimated. For instance, a prevalence of infection of only 7% was reported for *O. lauquen* in *Galaxias maculatus* (Jenyns) based on microscopic observations, but molecular analysis revealed this value to be significantly higher (49%) (Alama-Bermejo et al., 2019). Similarly, prevalence of infection of *O. concentrica* in *Acanthistius patachonicus* (Jenyns) was determined to be 30% and 60%, based on microscopic and molecular analyses, respectively (Alama-Bermejo & Hernández-Orts, 2018).

About one third of *Ortholinea* species (9/26) have the small subunit ribosomal RNA gene (SSU rDNA) sequences available in GenBank, most of which were obtained from infections in their type hosts and provided in the original descriptions. The only exception is *O. orientalis*, of which SSU rDNA sequences were provided from infections in fish other than the original host (Karlsbakk & Køie, 2011). Another 6 *Ortholinea* SSU rDNA sequences are available, but are unpublished records (DQ333433, KP637274, MH197371, MK937851, MZ474836, KU301052). A single large subunit ribosomal RNA gene (LSU rDNA) sequence is also available for *Ortholinea nupchi* Shin et al., 2023. Genetic interspecific variation between *Ortholinea* SSU rDNA sequences ranges between 1.65% and 29.1%. Phylogenetic analyses reveal the polyphyly of this genus, whose members cluster within the freshwater urinary clade of the oligochaete-infecting lineage, alongside the sequences of *Hoferellus* spp., *Myxobilatus gasterostei* (Parisi, 1912), *Acauda hoffmani* Whipps, 2011, *Myxidium giardi* Cépède, 1906, *Chloromyxum schurovi* Shulman & Ieshko, 2003, *Zschokkella* sp. ex *Anguilla anguilla*, *Myxidium streisingeri* Whipps, Murray & Kent, 2015, and the Triactinomyxon type of Rosser et al. (2014). This heterogeneous clade is composed by several distinct genera/myxospore morphologies, but also species infecting fish or amphibian hosts, either in freshwater or marine environments. The only features that unite the members of the freshwater urinary clade is their tropism to the urinary system, and the fact that they most likely use oligochaetes as invertebrate hosts (Holzer et al., 2018). This assumption is supported by the life cycles of *O. auratae* Rangel et al., 2014 and *O. labracis* Rangel et al., 2017, which involve oligochaetes as invertebrate hosts (Rangel et al., 2015, 2017).

Thus far, only these two *Ortholinea* species have their life cycle clarified, based on molecular inferences established between myxospore and actinospore counterparts (Rangel et al., 2015, 2017). *Ortholinea auratae* from the gilthead seabream *Sparus aurata* Linnaeus was shown to infect the oligochaete *Limnodriloides agnes* Hrabě (Rangel et al., 2015), while *O. labracis* from the European seabass *Dicentrarchus labrax* (Linnaeus) infects an unidentified oligochaete of the genus *Tectidrilus* (Rangel et al., 2017). Both oligochaete hosts belong to the family Naididae, and are distributed in estuarine and marine environments, with *L. agnes* typically occurring in subtidal sandy areas, and *Tectidrilus* in subtidal muddy sands (Erséus, 1982). The life cycles of *O. auratae* and *O. labracis* identify actinospores of the triactinomyxon collective group as counterparts for *Ortholinea*. This myxospore/actinospore correlation is further reinforced by the placement of the Triactinomyxon type of Rosser et al. (2014) within the freshwater urinary clade.

## Species Remarks

### ***Ortholinea africanus*** Abdel-Ghaffar, El-Toukhy, Al-Quraishy, Al-Rasheid, Abdel-Baki, Hegazy & Bashtar, 2008 (Fig. 1)

**Figure 1–14:**
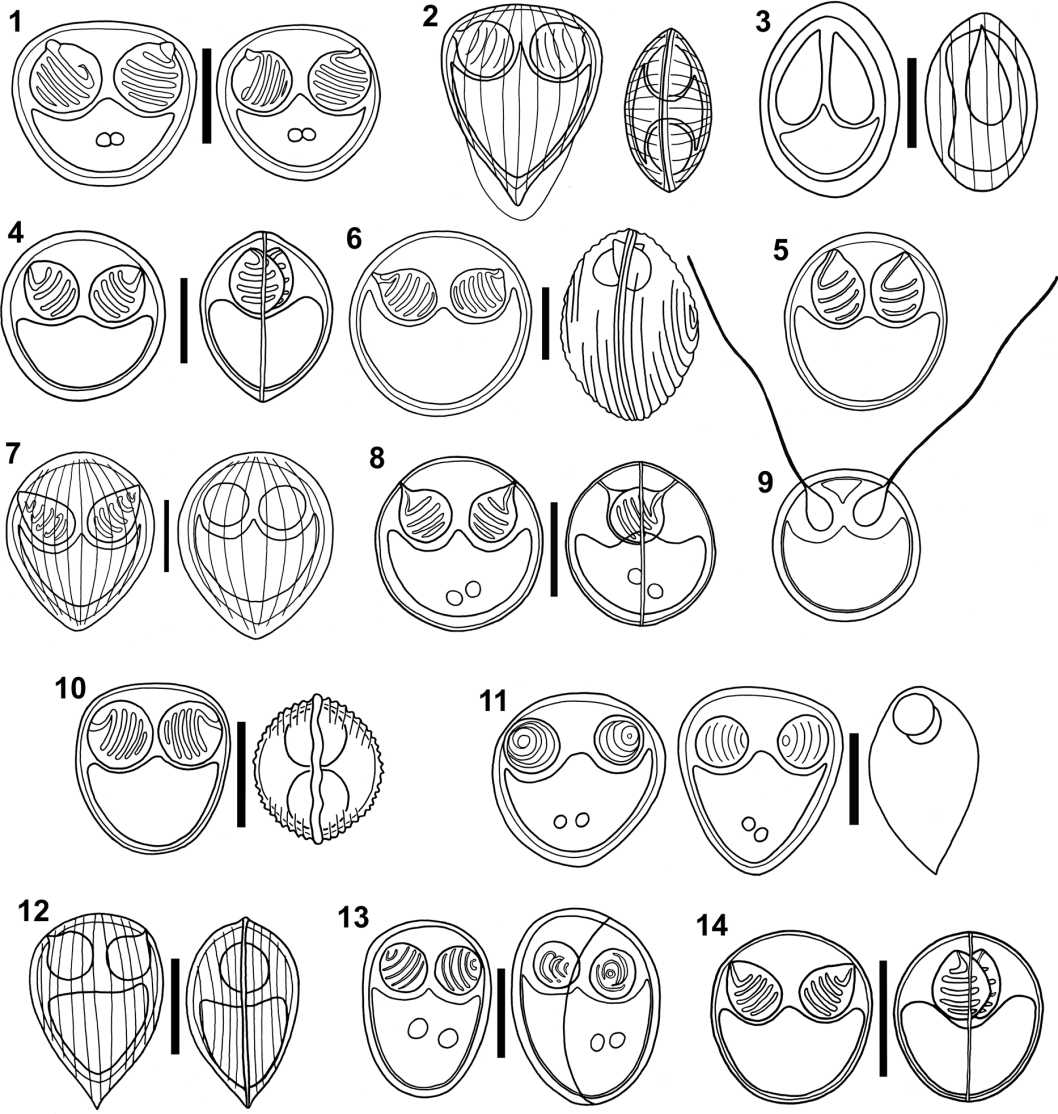
Line drawings of myxospores of *Ortholinea* spp. redrawn from the original illustrations. All scale-bars: 5 µm. 1) *Ortholinea africanus*; 2) *Ortholinea antipae*; 3) *Ortholinea asymmetrica*; 4) *Ortholinea auratae*; 5) *Ortholinea australis*; 6) *Ortholinea basma*; 7) *Ortholinea clupeidae*; 8) *Ortholinea concentrica*; 9) *Ortholinea divergens*; 10) *Ortholinea fluviatilis*; 11) *Ortholinea gadusiae*; 12) *Ortholinea gobiusi*; 13) *Ortholinea indica*; 14) *Ortholinea labracis*.

Freshwater. Originally reported from the urinary bladder of the Nile tilapia *Oreochromis niloticus* (Linnaeus) (Cichliformes, Cichlidae), in Bahr Shebin, Nile Delta, Egypt. Coelozoic, with disporic and polysporic plasmodia. Valves with 10 to 16 surface ridges. Prominent suture line. Iodinophilous vacuole present. Prevalence of infection of 44.7% (34/76) (Abdel-Ghaffar et al., 2008).

Ali (2009) redescribed *O. africanus* from its original host and geographic locality, using scanning electron microscopy to study the valve ornamentation of the myxospores (Table 2; Fig. 27). The author reported mono- to polysporic plasmodia, and myxospores with considerably bigger dimensions than those reported in the original description, without providing a reasoning for these differences.

**Table 2:** Data from *Ortholinea* species redescriptions. Myxospore measures are given in micrometres and, when available, include mean, standard deviation, and range in parentheses. Absence of data is indicated by a hyphen. Abbreviations: F, Figure number; PCL, Polar capsule length (or diameter when width is missing); PCW, Polar capsule width; PTC, Polar tubule coils; SBL, Myxospore length; SBT, Myxospore thickness; SBW, Myxospore width; *) Measurements from fixed spores; **) Diameter.

Species	F	SBL	SBW	SBT	PCL	PCW	PTC	Source
*O. africanus*	27	9.6 ± 0.8 (8.1–10.9)	9.5 ± 0.6 (8.8–10.9)	8.9 ± 0.4 (8.6–9.1)	3.9 ± 0.3 (3.0–4.3)	3.9 ± 0.3 (3.0–4.3)	4–6	Ali (2009)
*O. divergens*	28	10,0	–	8,0	4,0	–	–	Auerbach (1912)
O. divergens*	–	8.7 ± 0.3 (7.5–10.5)	**	7.0 ± 0.2 (6.5–8.0)	3.3 ± 0.1 (3.0–4.0)	–	6–8	Moser & Noble (1977)
*O. divergens*	29	9.2 ± 0.78 (8.0–10.0)	9.4 ± 0.39 (8.8–10.0)	5.1 ± 0.30 (4.8–5.4)	–	–	–	Wierzbicka (1990)
O. divergens*	–	8.3 ± 0.36 (8.0–8.8)	8.4 ± 0.34 (8.0–9.2)	5.9 ± 0.57 (4.8–6.8)	–	2.6 ± 0.26 (2.4–3.2)	–	Wierzbicka (1990)
*O. divergens*	–	9.0 (8.1–9.4)	9.2 (8.4–9.7)	–	2.0 (1.9–2.2)	2.2 (1.9–2.4)	–	Özer et al. (2015)
*O. orientalis*	–	8.5–11.5	6.8–9.8	6.6–8.0	3.0–4.2	–	–	Shulman & Shulman-Albova (1953)
*O. orientalis*	–	7.8	7.3	–	3.1	–	–	Shulman & Shulman-Albova (1953)
*O. orientalis*	30	7.3–9.0	6.3–7.2	–	2.8–3.2	1.8–2.0	–	Aseeva (2000)
*O. orientalis*	30	9.3–10.3	8.6–9.3	–	3.5–4	2.7–3.5	4	Aseeva (2002)
*O. orientalis*	30	7.6–8.3	6.6–8.0	–	3.0–3.7	3.0–3.7	5	Aseeva (2002)
*O. orientalis*	31	9.0 (8.5–9.2)	7.9 (7.7–8.0)	5.6 (4.9–5.8)	2.7 (2.3–2.9)	2.7 (2.3–2.9)	–	Karlsbakk & Køie (2011)
*O. polymorpha*	32	7.5–9.5	7.5–9.5	7.0–8.0	3.8–5.0	2.0–2.8	3–6	Kudo (1944)
O. sphaerocapsularae*	–	9.8 ± 0.7 (8.4–11.2)	8.8 ± 0.5 (8.0–10.0)*	–	3.7 ± 0.4 (3.2–4.4)	3.0 ± 0.3 (2.4–3.4)	6	Wierzbicka, 1986
*O. undulans*	–	8.2 (7.0–9.0)	7.4 (7.0–8.0)	6.2 (6.0–7.0)	3.1 (3.0)	2.5 (2.0–3.0)	–	Meglitsch (1970)

### ***Ortholinea antipae*** Moshu & Trombitsky, 2006 (Fig. 2)

Marine. In the urinary bladder, ureters, and lumen of the kidney tubules of the Black Sea shad *Alosa tanaica* (Grimm) (Clupeiformes, Alosidae) from the Sasyk lake and Cuciurgan Reservoir, Odessa Oblast, Ukraine. Coelozoic, with plasmodia disporic or polysporic (4 to 6 spores). Valves with 10 to 16 surface ridges. Inconspicuous suture line. Prevalence of infection of 34.8% (8/23) (Moshu & Trombitsky, 2006).

Moshu and Trombitsky (2006) suggested that *O. antipae* and *O. orientalis* might constitute geographic isolates of a single species, given that they share significant morphological similarities, differing solely in the presence/absence of valve ornamentation, respectively (Shulman & Shulman-Albova, 1953). The redescription of *O. orientalis* by Karlsbakk & Køie (2011) showed that the myxospores of this species actually have surface ridges, thus raising the possibility that *O. antipae* be a synonym of *O. orientalis*.

### ***Ortholinea asymmetrica*** Kovaleva, Velev & Vladev, 1993 (Fig. 3)

Marine. In the gall bladder of the False scad *Caranx rhonchus* Geoffroy Saint-Hilaire (Carangiformes, Carangidae) caught off the Atlantic coast of the Republic of Sierra Leone, Africa. Coelozoic, forming polysporic plasmodia containing 8 or more myxospores. Valves asymmetric due to shifted suture line and ornamented by surface ridges. Prevalence of infection of 13.3 % (2/15) (Kovaleva et al., 1993).

Original depictions by Kovaleva et al. (1993) show the myxospores of *O. asymmetrica* having polar capsules positioned parallel to each other in one drawing, and convergent in another drawing. This positioning of the polar capsules does not agree with the taxonomic features of *Ortholinea*, best representing those of the genus *Myxobolus*. However, it is unclear if this representation should be considered as the result of taxonomic misidentification, given that the histozoic nature of *Myxobolus* does not conform with the species report performed by Kovaleva et al. (1993). It is more likely that myxospores have been poorly represented in the original drawings, as was the case of *O. polymorpha*, originally drawn with parallel polar capsules by Davis (1917), and later redescribed with polar capsules opening to opposite sides by Kudo (1944). Thus, solving this issue will require the morphological redescription and molecular analyses of *O. asymmetrica* from its original host.

### ***Ortholinea auratae*** Rangel, Rocha, Borkhanuddin, Cech, Castro, Casal, Azevedo, Severino, Székely & Santos, 2014 (Fig. 4)

Marine. In the urinary bladder and terminal portion of the posterior kidney of the Gilthead seabream *Sparus aurata* Linnaeus (Eupercaria, Sparidae) from the Alvor estuary, Atlantic coast (37° 08′ N, 8° 37′ W), Portimão, Algarve, Portugal. Coelozoic, with polysporic, and less frequently disporic plasmodia. Valves with 19 or more surface ridges. Suture line straight and evident. Prevalence of infection of 51.6% (64/124) (Rangel et al., 2014).

SSU rDNA sequences from the original description deposited in GenBank (KF703856–KF703858, KR025868–KR025869).

The life cycle counterpart of *O. auratae* was molecularly inferred to be a triactinomyxon type developing in the intestinal epithelium of the marine oligochaete *Limnodriloides agnes* Hrabě (Tubificida, Naididae). Infection in the invertebrate host was detected in a Portuguese semi-intensive fish farm (Portimão, Portugal), with a prevalence of infection of 16.0% in the earth ponds containing *S. aurata* (Rangel et al., 2015).

### ***Ortholinea australis*** Lom, Rohde & Dyková, 1992 (Fig. 5)

Marine. In the gall bladder and biliary ducts of the Yellowfin bream *Acanthopagrus australis* (Günther) (Eupercaria, Sparidae) from Coffs Harbour, New South Wales, Australia. Also described from the Goldlined seabream *Rhabdosargus sarba* (Forsskål) (Eupercaria, Sparidae). Coelozoic, with polysporic plasmodia. Valves ornamented by surface ridges. Suture line straight and evident. Prevalence of infection of 16.6% (2/12) for *A. australis* and of 25% (3/12) for *R. sarba* (Lom et al., 1992).

### ***Ortholinea basma*** Ali, 2000 (Fig. 6)

Marine. In the urinary bladder of the Agile klipfish *Clinus agilis* Smith, 1931 (Blenniiformes, Clinidae) from Port Nolloth, West coast, South Africa. Coelozoic, with mono-, di- (rarer) and polysporic plasmodia. Valves with 12 to 13 surface ridges. Prevalence of infection of 16.6% (2/12) (Ali, 2000).

### ***Ortholinea clupeidae*** Aseeva, 2000 (Fig. 7)

Marine. In the gall bladder and urinary tubules of the Pacific herring *Clupea pallasii* Valenciennes (Clupeiformes, Clupeidae) and the Dotted gizzard shad *Konosirus punctatus* (Temminck & Schlegel) (Clupeiformes, Dorosomatidae) from Stark strait and Serebryanka Bay, Amur Bay, in Primorsky Krai, Russia. Coelozoic, with polysporic plasmodia containing 6 to 10 myxospores. Valves ornamented by surface ridges. Suture line thin and protruding. Prevalence of infection of 4.2% (2/48) in *C. pallasi* and of 50.0% (1/2) in *K. punctatus* (Aseeva, 2000).

Karlsbakk and Køie (2011) suggested *O. clupeidae* be considered a synonym of *O. orientalis*, based on the unreliability of using the presence/absence of surface ridges for species differentiation.

### ***Ortholinea concentrica*** Alama-Bermejo & Hernández-Orts, 2018 (Fig. 8)

Marine. In the urinary bladder, kidney, and ureters of the Patagonian seabass *Acanthistius patachonicus* (Jenyns) (Perciformes/Serranoidei, Anthiadidae) from Punta Verde, San Antonio Bay, San Matías Gulf (40° 43′ 47″ S, 64° 54′ 45″ W) Rio Negro, Argentina. Coelozoic, with polysporic plasmodia. Valves with 17 to 20 surface ridges. Suture line straight and transverse. Prevalence of infection of 30.0% (3/10) based on microscopic observations, and of 60.0% (6/10) based on molecular screening (Alama-Bermejo & Hernández-Orts, 2018).

SSU rDNA sequences from the original description deposited in GenBank (MH793343–MH793353).

### ***Ortholinea divergens*** (Thélohan, 1895) Shulman, 1962 (Fig. 9)

Synonym: *Sphaerospora divergens* Thélohan, 1895

Marine. In the kidney tubules of the Shanny *Lipophrys pholis* (Linnaeus) (Blenniiformes, Blenniidae) and the Corkwing wrasse *Symphodus melops* (Linnaeus) (Eupercaria, Labridae) from Roscoff and Concarneau, Bretagne, France. Coelozoic, with polysporic plasmodia. Valves ornamented by surface ridges. Prevalence of infection in *L. pholis* 14.3% (1/7) in Roscoff and 33.3% (1/3) in Concarneau. Prevalence of infection in *S. melops* 4.4% (1/23) in Roscoff and 8.3% (1/12) in Concarneau (Thélohan, 1895).

Several redescriptions of *O. divergens* can be found in the literature. Auerbach (1912) redescribed *O. divergens* from the urinary bladder of the American plaice *Hippoglossoides platessoides* (Fabricius) (Pleuronectiformes; Pleuronectidae) from Tanafjord, Norway (Table 2; Fig. 28). Moser and Noble (1977) redescribed *O. divergens* from the gall bladder of the Hollowsnout grenadier *Coelorinchus caelorhincus* (Risso) (Gadiformes; Macrouridae) from the coast of the Republic of Suriname (Table 2). Wierzbicka (1990) redescribed *O. divergens* from the urinary bladder of the Greenland halibut *Reinhardtius hippoglossoides* (Walbaum) (Pleuronectiformes; Pleuronectidae) from the North Atlantic, Barents Sea (Table 2; Fig. 29). Finally, Özer et al. (2015) redescribed *O. divergens* from the urinary bladder of the Rusty blenny *Parablennius sanguinolentus* (Pallas) (Blenniiformes; Blenniidae) from Sinop, Black Sea, Turkey (Table 2). There are also many other reports of this species in parasite surveys of fish: in the kidney of the East Atlantic peacock wrasse *Symphodus tinca* (Linnaeus) (Eupercaria; Labridae) from Napoli, Italy (Parisi, 1912); in the urinary bladder of the Hornyhead turbot *Pleuronichthys verticalis* Jordan & Gilbert (Pleuronectiformes; Pleuronectidae) from Southern California, USA (Jameson, 1931); in the American plaice *H. platessoides* and the Greenland halibut *R. hippoglossoides* from the Northwest Atlantic region (Zubchenko, 1980); and in the urinary bladder of the Striped seaperch *Embiotoca lateralis* Agassiz and the Pile perch *Phanerodon vacca* (Girard) (Ovalentaria; Embiotocidae) from Avila Beach, California, USA and Santo Tomás, Mexico (Moser & Haldorson, 1982).

### ***Ortholinea fluviatilis*** Lom & Dyková, 1995 (Fig. 10)

Freshwater. In the kidney ducts and tubules of the Green pufferfish *Dichotomyctere fluviatilis* (Hamilton) imported from Southeast Asia to the Czech Republic. Coelozoic, with polysporic plasmodia. Valves ornamented by surface ridges. Suture line slightly undulated. Prevalence of infection of 100% (7/7) (Lom & Dyková, 1995).

### ***Ortholinea gadusiae*** Sarkar, 1999a (Fig. 11)

Marine. In the urinary bladder of the Indian river shad *Gudusia chapra* (Hamilton) (Clupeiformes, Dorosomatidae) from the Bay of Bengal near Digha, West Bengal, India. Coelozoic, with polysporic plasmodia. Valves smooth. Suture line thin and curved. Prevalence of infection of 3.6% (1/28) (Sarkar, 1999a).

### ***Ortholinea gobiusi*** Naidenova, 1968 (Fig. 12)

Marine. In the urinary bladder of the Grass goby *Gobius ophiocephalus* Pallas (Gobiiformes, Gobiidae) from Sevastopol, Black Sea, Crimea, Ukraine. Coelozoic, with disporic plasmodia. Valves ornamented by surface ridges. Suture line evident. Prevalence of infection not provided (Naidenova, 1968).

### ***Ortholinea indica*** Sarkar, 1999b (Fig. 13)

Marine. In the urinary bladder and kidneys of the Cuja bola *Macrospinosa cuja* (Hamilton) (Eupercaria, Sciaenidae) in South 24 Parganas district, West Bengal, India. Coelozoic, with disporic to polysporic plasmodia. Valves smooth. Suture line thin, curved, and inconspicuous. Prevalence of infection of 18.8% (3/16) (Sarkar, 1999b).

### ***Ortholinea labracis*** Rangel, Rocha, Casal, Castro, Severino, Azevedo, Cavaleiro & Santos, 2017 (Fig. 14)

Marine. In the urinary bladder and terminal portion of the posterior kidney of the European seabass *Dicentrarchus labrax* (Linnaeus) (Eupercaria, Moronidae) from the Alvor estuary, near the Atlantic coast (37° 08′ N, 08° 37′ W), Portimão, Algarve, Portugal. Coelozoic, with polysporic, and more rarely disporic plasmodia. Valves ornamented by surface ridges. Straight suture line. Prevalence of infection of 11.0% (17/155) (Rangel et al., 2017).

SSU rDNA sequences from the original description deposited in GenBank (KU363830–KU363831).

The life cycle counterpart of *O. labracis* was molecularly inferred to be a triactinomyxon type developing in the intestinal epithelium of the marine oligochaete *Tectidrilus* sp. (Tubificida, Naididae). Infection in the invertebrate host was detected in European seabass earth ponds of a Portuguese semi-intensive fish farm (Portimão, Portugal), with a prevalence of infection of 9.5% (18/190) (Rangel et al., 2017).

### ***Ortholinea lauquen*** Alama-Bermejo, Viozzi, Waicheim, Flores & Atkinson, 2019 (Fig. 15)

Freshwater. In the kidney tubules of the Inanga *Galaxias maculatus* (Jenyns) (Galaxiiformes, Galaxiidae) from Lago Moreno (41° 3′ 34.67″ S, 71° 33′ 50.82″ W), San Carlos de Bariloche, Río Negro, Argentina. Coelozoic, with disporic to polysporic plasmodia (up to 6 myxospores per plasmodium). Valves with 15 to 20 surface ridges. Straight suture line. Prevalence of infection of 7.0% (8/114) based on microscopic observations, and of 49.0% (49/100) based on molecular screening (Alama-Bermejo et al., 2019).

**Figure 15–26:**
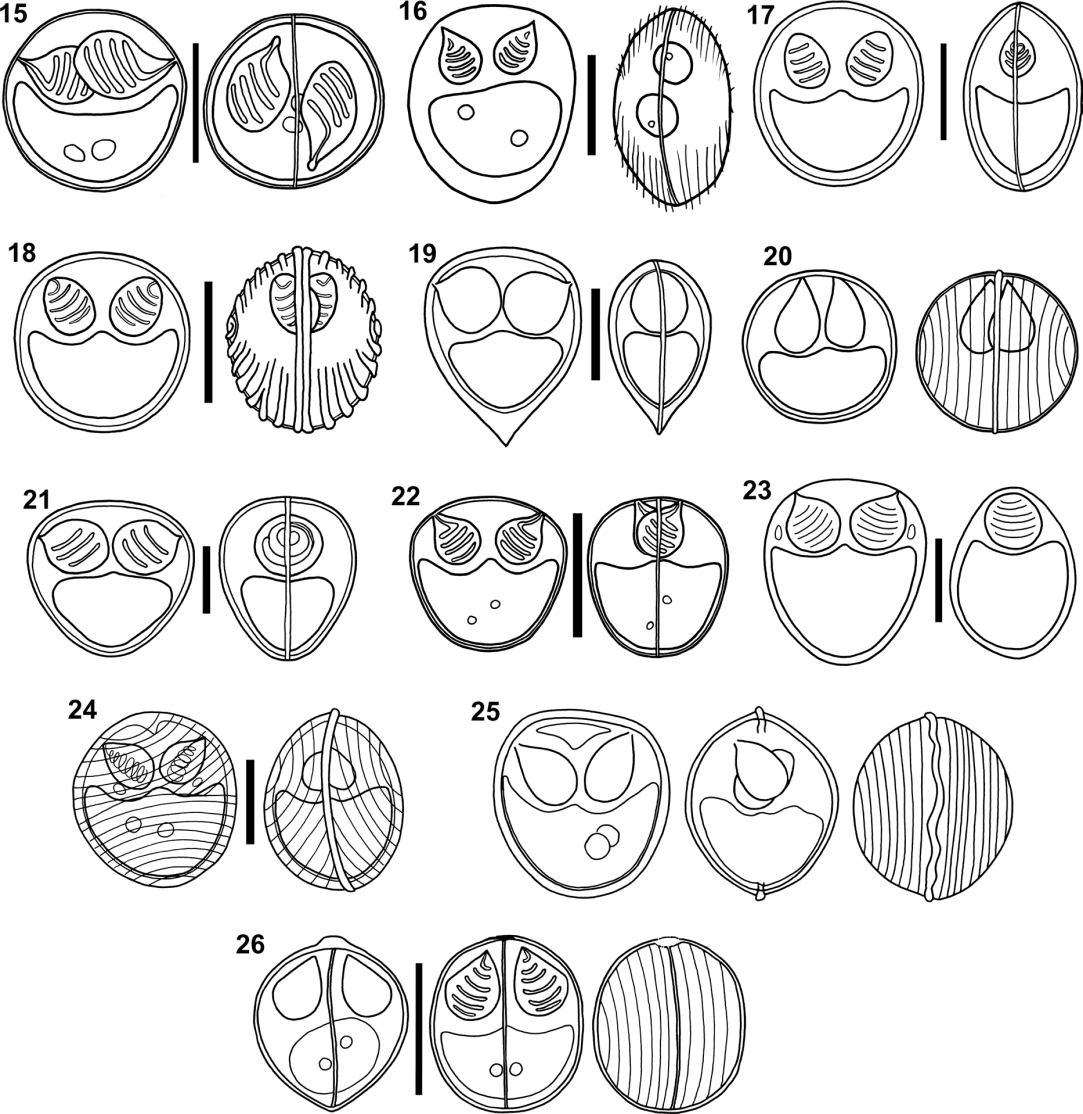
Line drawings of myxospores of *Ortholinea* spp. redrawn from the original illustrations. All scale-bars: 5 µm. 15) *Ortholinea lauquen*; 16) *Ortholinea macrouri*; 17) *Ortholinea mullusi*; 18) *Ortholinea nupchi*; 19) *Ortholinea orientalis*; 20) *Ortholinea polymorpha*; 21) *Ortholinea saudii*; 22) *Ortholinea scatophagi*; 23) *Ortholinea sphaerocapsularae*; 24) *Ortholinea striateculus*; 25) *Ortholinea undulans*; 26) *Ortholinea visakhapatnamensis*.

SSU rDNA sequences from the original description deposited in GenBank (MN128723–MN128729).

### ***Ortholinea macrouri*** Kovaleva, Velev & Vladev, 1993 (Fig. 16)

Marine. In the urinary bladder of the banded whiptail *Coelorinchus fasciatus* (Günther) (Gadiformes, Macrouridae) from the coast of Namibia. Coelozoic, with polysporic plasmodia. Valves ornamented by surface ridges. Straight suture line. Prevalence of infection not provided (Kovaleva et al., 1993).

### ***Ortholinea mullusi*** Gürkanlı, Okkay, Çiftçi, Yurakhno & Özer, 2018 (Fig. 17)

Marine. In the urinary bladder and kidney tubules of the red mullet *Mullus barbatus* Linnaeus (Mulliformes, Mullidae) from the coast of Sinop, Black Sea (42° 02′ 51″ N, 35° 02′ 56″ E), Turkey. Coelozoic, with polysporic plasmodia. Valves ornamented by surface ridges. Prevalence of infection of 24.5% (49/200) (Gürkanlı et al., 2018).

SSU rDNA sequence from the original description deposited in GenBank (MF539825).

### *Ortholinea nupchi* Shin, Jin, Sohn, Kim & Lee, 2023 (Fig. 18)

Marine. In the urinary bladder of the Bastard halibut *Paralichthys olivaceus* (Temminck & Schlegel) (Pleuronectiformes, Paralichthyidae) from a fish farm in Jeju Self-Governing Province (33° 15′ N, 126° 11′ E), Republic of Korea. Vegetative development not described. Valves with 5 to 7 surface ridges. Suture line straight and evident. Prevalence of infection of 50.0% (5/10) (Shin et al., 2023).

Original description with SSU rDNA (MW540886) and LSU rDNA (MW540892) sequences deposited in GenBank.

### ***Ortholinea orientalis*** (Shulman & Shulman-Albova, 1953) Shulman, 1962 (Fig. 19)

Synonym: *Sphaerospora orientalis* Shulman & Shulman-Albova, 1953

Marine. In the urinary bladder of the Pacific herring *Clupea pallasii* Valenciennes (Clupeiformes, Clupeidae) (Table 1), and in the urinary bladder and gall bladder (more rarely) of the gadid fishes, the Saffron cod *Eleginus gracilis* (Tilesius) and the Navaga *Eleginus nawaga* (Walbaum) from the White Sea, Russia (Table 2). The authors further refer the Pacific Ocean as another locality where this parasite is present. Coelozoic, with disporic to polysporic (2 to 10 myxospores) plasmodia. Valves without ornamentation. Prevalence of infection of 28.6% (2/7) (Shulman & Shulman-Albova, 1953).

Aseeva (2000) redescribed *O. orientalis* from the urinary bladder and kidneys of *C. pallasii* from the Sea of Japan, Primorsky Krai, Russia, with a prevalence of infection of 29.2% (14/48). Another redescription was performed by Aseeva (2002), from the urinary bladder and kidneys of the Alaska pollock *Gadus chalcogrammus* Pallas and *E. gracilis* from southwestern Kamchatka (Sea of Okhotsk), western part of the Bering Sea, Russia (Table 2; Fig. 30).

**Figure 27–32:**
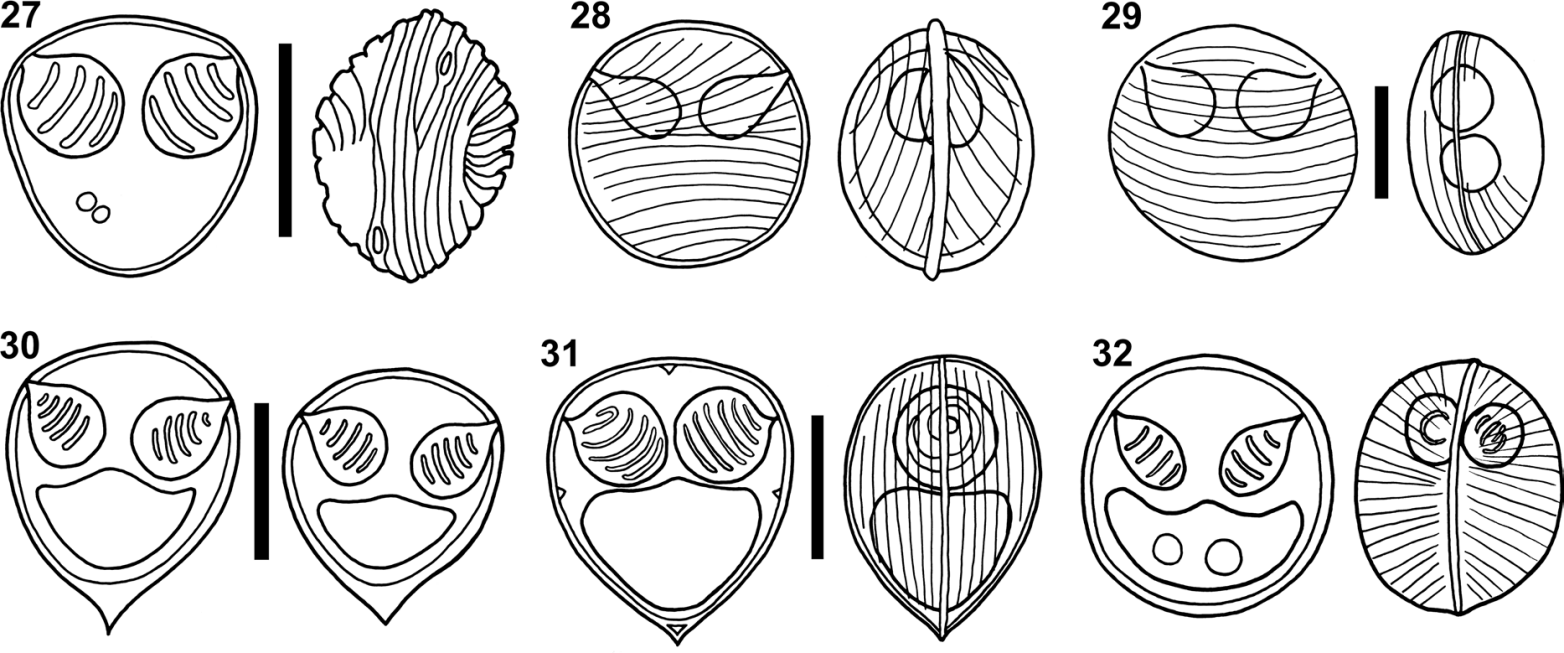
Line drawings of redescribed myxospores of *Ortholinea* spp. redrawn from the original illustrations. All scale-bars: 5 µm. 27) *Ortholinea africanus* from Ali, 2009; 28) *Ortholinea divergens* from Auerbach, 1912; 29) *Ortholinea divergens* from Wierzbicka, 1990; 30) *Ortholinea orientalis* from Aseeva 2000, 2002; 31) *Ortholinea orientalis* from Karlsbakk & Køie, 2011; 32) *Ortholinea polymorpha* from Kudo, 1944.

According to Karlsbakk and Køie (2011), the original description of *O. orientalis* is not correct, given that the methodology (glycerin-gelatine) used by Shulman and Shulman-Albova (1953) causes myxospores to shrink, further hindering perception of important details like the presence/absence of valve ornamentations. These authors redescribed *O. orientalis* from the ureters of the Atlantic herring *Clupea harengus* Linnaeus and the European sprat *Sprattus sprattus* (Linnaeus) from Øresund, Denmark. Myxospores were redescribed having valves ornamented by surface ridges, one intercapsular process, and two sutural edge markings (Table 2; Fig. 31), and *C. pallasi* was suggested as type host, and the White Sea as type locality. The authors further considered *O. antipae* and *O. clupeidae* to be synonyms of *O. orientalis*, given that the main character differentiating between these species is the presence/absence of valve ornamentations.

The SSU rDNA sequences of *O. orientalis* available in GenBank (HM770871–HM770875) were provided by Karlsbakk and Køie (2011) from infections in *C. harengus* and *S. sprattus*.

### ***Ortholinea polymorpha*** (Davis, 1917) Shulman, 1962 (Fig. 20)

Synonym: *Sphaerospora polymorpha* Davis, 1917

Marine. In the urinary bladder of the Oyster toadfish *Opsanus tau* (Linnaeus) (Batrachoidiformes, Batrachoididae) from Beaufort, North Caroline, USA. Coelozoic, with disporic to polysporic plasmodia. Valves ornamented by surface ridges. Suture line evident. Prevalence of infection of 81.8% (9/11) (Davis, 1917).

Kudo (1944) redescription of *O. polymorpha* corrected the positioning of the polar capsules, which according to the drawings of Davis (1917) were parallel to each other. The myxospores were redescribed based on fresh and fixed material collected from the type host *O. tau* from the Solomon Islands, Maryland, USA, and from the Gulf toadfish *Opsanus beta* (Goode & Bean) from Englewood, Florida, USA (Table 2; Fig. 32). Both hosts showed prevalences of infection of 100%.

### ***Ortholinea saudii*** Abdel-Baki, Soliman, Saleh, Al-Quraishy & El-Matbouli, 2015 (Fig. 21)

Marine. In the kidneys of the Marbled spinefoot *Siganus rivulatus* Forsskål & Niebuhr (Acanthuriformes, Siganidae) from the Jeddah Red Sea coast (21° 31′ N, 39° 13′ E), Saudi Arabia. Coelozoic. Valves smooth. Suture line indistinct. Prevalence of infection of 5.0% (2/40) (Abdel-Baki et al., 2015).

SSU rDNA sequence from the original description deposited in GenBank (JX456461).

### ***Ortholinea scatophagi*** Chandran, Zacharia & Sanil, 2020 (Fig. 22)

Marine. In the urinary bladder and ureter of the Spotted scat *Scatophagus argus* (Linnaeus) (Acanthuriformes, Scatophagidae) from Cochin (9° 59.001′ N; 76° 14.584′ E), Southwest coast of India. Coelozoic, with mono-, di- to polysporic plasmodia. Valves with 15 to 19 surface ridges. Suture line straight and prominent. Prevalence of infection of 70.1% (249/355) (Chandran et al., 2020).

SSU rDNA sequence from the original description deposited in GenBank (MN310514).

### ***Ortholinea sphaerocapsularae*** (Wierzbicka, 1986) Sitjà-Bobadilla & Álvarez-Pellitero, 1994 (Fig. 23)

Synonym: *Sphaerospora sphaerocapsularae* Wierzbicka, 1986

Freshwater. In the urinary bladder of the European eel *Anguilla anguilla* (Linnaeus) (Anguilliformes, Anguillidae), from Lake Dąbie, Poland. Coelozoic. Valves ornamented by surface ridges. Suture line straight, inconspicuous. Prevalence of infection of 7.7% (1/13) (Wierzbicka, 1986).

Wierzbicka (1986) described *O. sphaerocapsularae* as a member of the genus *Sphaerospora*, which explains for the swap between myxospore width and thickness in the original description. It is possible that infection by *O. sphaerocapsularae* is acquired in marine or estuarine environments, given that the European eel is a catadromous species that reproduces in the Sargasso Sea, with larval stages migrating to European rivers to grow into adults (Wright et al., 2022).

### ***Ortholinea striateculus*** Su & White, 1994 (Fig. 24)

Marine. In the ureter of the Silver fish *Leptatherina presbyteroides* (Richardson) (Atheriniformes, Atherinidae) from Dru Point, North-West Bay, Tasmania, Australia. Coelozoic. Valves with 18 to 20 surface ridges. Suture line straight and evident. Prevalence of infection of 0.3% (2/589) (Su & White, 1994).

### ***Ortholinea undulans*** (Meglitsch, 1970) Arthur & Lom, 1985 (Fig. 25)

Synonym: *Sphaerospora undulans* Meglitsch, 1970

Marine. In the urinary bladder, ureters and inferior part of the oviducts of the Witch *Arnoglossus scapha* (Forster) (Pleuronectiformes, Bothidae) from Wellington and Napier, New Zealand. Also described from the New Zealand sole *Peltorhamphus novaezeelandiae* Günther (Pleuronectiformes, Rhombosoleidae). Coelozoic, with polysporic plasmodia. Valves with ca. 20 surface ridges. Suture line undulated and prominent. Prevalence of infection not provided (Meglitsch, 1970).

### ***Ortholinea visakhapatnamensis*** Padma Dorothy & Kalavati, 1993 (Fig. 26)

Marine. In the visceral peritoneum of the Largescale mullet *Planiliza macrolepis* (Smith) (Mugiliformes, Mugilidae) from Visakhapatnam, Andhra Pradesh, Bay of Bengal (17° 41′ 34″ N, 83° 17′ 35″ E), India. Histozoic, with cyst formation. Valves with 8 surface ridges. Straight suture line. Prevalence of infection of 20.3% (Padma Dorothy & Kalavati, 1993).

## Data Availability

Not applicable.
